# The slow decay and quick revival of self-deception

**DOI:** 10.3389/fpsyg.2015.01075

**Published:** 2015-08-19

**Authors:** Zoë Chance, Francesca Gino, Michael I. Norton, Dan Ariely

**Affiliations:** ^1^Yale School of Management, Yale University, New Haven, CT, USA; ^2^Harvard Business School, Harvard University, Boston, MA, USA; ^3^Fuqua School of Business, Duke University, Durham, NC, USA

**Keywords:** self-deception, cheating, self-enhancement, positive illusions, motivated reasoning

## Abstract

People demonstrate an impressive ability to self-deceive, distorting misbehavior to reflect positively on themselves—for example, by cheating on a test and believing that their inflated performance reflects their true ability. But what happens to self-deception when self-deceivers must face reality, such as when taking another test on which they cannot cheat? We find that self-deception diminishes over time only when self-deceivers are repeatedly confronted with evidence of their true ability (Study 1); this learning, however, fails to make them less susceptible to future self-deception (Study 2).

## Introduction

Imagine a stock trader who has access to insider information on particular firms, and as a result of using this information earns exceptionally high returns. If he then judges his stock trading ability by this performance, he may deceive himself into expecting high returns when he invests in other firms as well—discounting his cheating as the cause of his performance in favor of a self-deceptive view that the performance was due to his ability. Imagine that in his subsequent trades, he is lacking any insider information; over time, as a result, his future portfolio performance will give him unbiased evidence of his true ability. Will the trader eventually readjust his self-deceptive beliefs, and come to a more realistic understanding of his true ability?

We study both the decay and subsequent revival of self-deception in situations in which cheaters who have believed their superior performance was due to exceptional ability are then confronted with evidence of their true ability. How many doses of reality does it take before the truth sinks in and is accepted? After realizing the force and pitfalls of self-deception, are individuals less likely to engage in self deception in the future?

### Motivated Views of the Self

People tend to see themselves through rose-tinted glasses. Decades of research document the tendency to self-enhance (Greenwald, 1980; Sedikides and Strube, 1997), with most people inflating their standing on positive attributes ranging from intelligence to ability to morality (Alicke, 1985; Taylor and Brown, 1988). Much of the empirical work on biased self-evaluations has explored the motivation for overestimating our own abilities or viewing ourselves as better than we truly are (e.g., Burson et al., 2006). This motivation is so strong that most people ignore or rationalize negative information about themselves to maintain a positive self-image (Pyszczynski and Greenberg, 1987; Kunda, 1990; Chance and Norton, 2010). They use motivated reasoning to interpret ambiguous information in ways that confirm their—generally positive—beliefs and attitudes about themselves (e.g., Lord et al., 1979; Ditto and Lopez, 1992; Swann et al., 1992). Moreover, people display impressive creativity in justifying questionable behavior and decisions (e.g., Norton et al., 2004; Gino and Ariely, 2012).

### Self-deception

Although honesty is central to the self-concept (Blasi, 1984; Aquino and Reed, 2002), people routinely attempt to deceive others: in one diary study, participants reported lying once or twice a day (DePaulo et al., 1996). While some of these are “white” lies to protect others’ feelings, many are self-serving. Rather than lying to maximize their economic utility, however, people often use a “fudge factor” that gives them some moral wiggle room—to lie or cheat just a little (Mazar et al., 2008). Farrington and Kidd (1977) show that people are more likely to dishonestly accept a smaller amount of money, and Goldstone and Chin (1993) show that people rarely fail to report making copies, but rather often underreport the number of photocopies they had made—even when they were not monitored.

Deceiving others has the potential benefit of getting ahead, even just to save a few pennies. But why would humans deceive themselves? Evolutionary psychologists have posited that self-deception evolved to assist in other-deception—the surest way to deceive others and not display signs of lying is to deceive oneself (e.g., Trivers, 2000). Most relevant to the present research, self-deception can allow people to hold preferred beliefs, regardless of the truth. Whereas motivated reasoning describes the general process of maintaining preferred beliefs, self-deception is a special case. “Stock examples of self-deception, both in popular thought and in the literature, feature people who falsely believe—in the face of strong evidence to the contrary—that their spouses are not having affairs, or that their children are not using illicit drugs, or that they themselves are not seriously ill” (Mele, 2001, p. 9). We follow Mele in defining self-deception as a positive belief about the self that persists in spite of disconfirming evidence.

Such beliefs can be maintained by attending to desirable evidence and avoiding conflicting undesirable evidence whenever possible. Greenwald (1997) compares knowledge avoidance to junk mail processing: if knowledge can be identified as unwelcome, a person may discard it before examining it thoroughly to learn precisely what it is. Self-deception is thus possible when ambiguity or vagueness leaves room for error or distortion (Gur and Sackeim, 1979; Baumeister, 1993; Mijovic-Prelec and Prelec, 2010; Sloman et al., 2010).

Chance et al. (2011) provided a new paradigm for demonstrating self-deception: participants who had an opportunity to cheat on a test by being given access to an answer key—and who therefore performed well—systematically overestimated their performance on future tests. Faced with the choice between attributing their performance to the presence of the answers or their own ability, people chose to self-deceive, convincing themselves that their performance was due not to the answers but to themselves. Importantly, Chance et al. (2011) incentivized participants for accurate predictions in one experiment. Whereas monetary incentives have eliminated face-saving lies in other studies (e.g., Dana et al., 2006), participants in the Chance et al. (2011) study who were paid for both performance and accuracy overpredicted their scores even when those overpredictions were costly—suggesting that overpredictions were self-deceptive rather than simply a no-consequence decision that allowed them to maintain consistency. As further evidence that the paradigm captures self-deception, overpredictions in the Chance et al. (2011) paradigm were correlated with trait self-deception, as measured by a scale of self-deceptive denial (Paulhus, 1998).

### The Present Research

Previous experiments have examined self-deception as a momentary phenomenon. Life, however, offers many opportunities to act, to gather information, and to update beliefs—or not. In this work, we allow participants to cheat on an ability-based task to reap greater financial reward. We suggest that, rather than interpreting their behavior as a negative signal about themselves (“I’m a cheater”), self-deceivers use the positive outcome of cheating to bolster positive beliefs about themselves (“I’m a high achiever”). We add to the previous research on self-deception by using a modified version of the paradigm developed by Chance et al. (2011) to study whether and how quickly self-deception decays when individuals are confronted with repeated evidence of their actual ability. Building on the previous work, we also test how people’s chronic tendencies to lie to themselves, and to others, relates to the pattern of overpredictions over time. Study 1 observes the decay of self-deception when an initial act of self-deception (inflating one’s sense of one’s abilities on the basis of a high score achieved by cheating) is followed by two rounds of unbiased feedback (scores on subsequent tests without an opportunity to cheat). Study 2 explores whether a second cheating opportunity can counteract the debiasing effect of feedback on actual abilities and reinstate self-deception. Together, these studies map the slow decay and quick revival of self-deception.

## Study 1: The Decay of Self-deception

Study 1 examines the extent to which self-deception persists despite repeated evidence against a desired self-view. Participants completed a battery of four tests of general knowledge, predicting their score before the last three. Some participants—those in the answers condition—had access to an answer key for Test 1, and we expected them to use it to cheat (evidenced by outperforming a control group without answers). We also expected their high scores to trigger self-deception, leading them to overpredict their scores on subsequent tests for which they did not have answer keys. Performance on these subsequent tests offered repeated evidence of participants’ true ability. We assessed the extent to which the inflated predictions of participants given the answers on Test 1 would be tempered by their later experience taking tests without the answers, hypothesizing that their predictions would eventually but not immediately converge with their true ability. Previous research has shown self-deception in this paradigm tracked with participants’ chronic inclination to self-deceive (Chance et al., 2011), and we expected that the decay of self-deception here would be related to chronic self-deception as well. We hypothesized that for participants in the answers condition, self-deception would be greater and persist longer for those who were dispositionally high in self-deception. Furthermore, using an other-deception related scale in combination with the self-deception scale allowed us to test whether prediction gaps were indeed correlated with self-deception and not with lying.

Since the design of these self-deception studies makes cheating ambiguous—intentionally so, to make self-deception possible—we conducted a pilot study to test whether using the answer key did indeed constitute cheating. According to Jones (1991) definition of unethical behavior, community members, rather than researchers or participants given the opportunity to cheat, are the appropriate judges of which behaviors constitute cheating. Sixty-five participants from Amazon’s Mechanical Turk read a description of our experimental research paradigm, including the instructions to participants, learned the results, and were asked to write four words describing the test takers. In their open-ended responses, “cheating” was the second most common open-ended response (15 people), after “dishonest” (22 people); 86% used the words “cheating,” “dishonest,” “unethical,” or synonyms of these words. Participants also rated the extent to which they considered this behavior to constitute cheating, on a 10-point scale (1: *definitely not cheating* to 10: *definitely cheating*). The mean response was 6.98 (SD = 2.86), with a modal response of “10.” Another group of 64 participants read about participants in the control condition, and indicated on the same scale whether that group was cheating; the mean response was 2.50 (SD = 2.63); the modal response was “1” (definitely not cheating). These results suggest that people judge the behavior of study participants in the answers condition who achieve higher scores to be unethical, such that “cheating” is an appropriate descriptor of their behavior. Cheaters do not need to perceive themselves as cheaters—indeed, they may be self-deceived.

### Materials and Methods

#### Participants

Seventy-one student and community member participants (33 male, *M*_*age*_ = 23.9, SD = 3.54) from the paid subject pool of a large, northeastern university were paid $20 to complete this experiment as the first of a series of unrelated studies during a 1-h group lab session. Participants also had the opportunity to earn performance-based bonus pay. Sample size was determined by laboratory capacity, and privacy dividers separated participants from one another.

#### Design and Procedure

Each participant was assigned to either the control or the answer condition. Both groups completed a series of four tests of general knowledge trivia, such as “What is the only mammal that truly flies?” (Moore and Healy, 2008), configured into four 10-question tests. Participants learned at the beginning of the study that in all four tests, they would earn a $0.25 bonus for each correct answer. This incentive encourages cheating, which is required for self-deception in this paradigm, although a monetary incentive is not always necessary for prompting cheating and self-deception (Chance et al., 2011).

For Test 1, participants in the answers condition had the answers to all ten questions printed in an answer key at the bottom of the page. Their instructions read, “It’s okay to check your answers as you go, but please do your own work.” These instructions were intentionally ambiguous—they did not prohibit looking at the answers, but they did imply that using the answer key to choose answers would be wrong. The control group completed the same test questions but without the answer key or instructions. All participants were given 3 min to complete Test 1. After handing their completed Test 1 to an experimenter, they were given a score sheet with an answer key, on which they recorded from memory which questions they had answered correctly. This procedure prevented participants in the control group from using the answer key to change their answers. It did not prevent either group from inflating their reported score, therefore we recorded the actual score as well. After completing and turning in the score sheet, participants in both conditions had seen the answers for Test 1 and knew their Test 1 scores.

When participants received Test 2, they were asked to look it over before writing down their predicted score. The preview ensured that those in the answers group could confirm that the test would not include an answer key. It also reduced the implicit admission of guilt that might be associated with predicting a lower score on the second test than the first (“If I say I will do worse, the researchers will know I cheated”), by giving participants a valid excuse (“I just don’t happen to know these particular answers”). Thus, this design provided a strong test of our prediction that participants who had cheated on the first test would deceive themselves into predicting an unrealistically high score on the second.

After predicting their score, participants spent 3 min completing Test 2, then repeated the process three more times: scoring Test 2 on a separate answer sheet; looking over Test 3 and making a prediction; scoring Test 3 on a separate answer sheet; looking over Test 4 and making a prediction; and scoring Test 4 on a separate answer sheet. Note that for all participants, Tests 2, 3, and 4 did not include answers at the bottom; and participants had only one sheet in front of them (either a test/prediction sheet or an answer key/score sheet) at all times.

When participants had finished the testing procedure, they moved on to other unrelated studies which also included the Balanced Inventory of Desirable Responding (Paulhus, 1998). We used the self-deceptive enhancement and the impression management components of the BIDR, to distinguish dispositional self-deception from dispositional lying. At the end of the study session, participants received their bonus payment. Because participants were not deceived (by the experimenters), the university Human Subjects Committee approving the experiment determined that debrief was not required.

### Results and Discussion

#### Cheating

We predicted participants in the answers condition would inflate their performance on the first test by looking at the answers. Indeed, they reported scoring higher than the control group, *t*(69) = 6.62, *p* < 0.001, *d* = 1.58 (Table 1). Our subsequent analyses reflect reported scores, since self-deception relies on beliefs; however, using actual scores here or in any of the subsequent analyses did not affect the direction or significance of the results.

**Table 1:** **Study 1 scores and predictions**.

		**Test 1**	**Test 2**	**Test 3**	**Test 4**
Answers	Prediction		6.28	5.72	5.53
	Score	7.89*	4.94	5.06	5.11
Control	Prediction		5.46	5.06	5.03
	Score	4.51	5	4.77	4.86

*Answer key available, cheating possible.

On the test in which cheating was possible, the average score was 7.89 out of 10, indicating either a mixture of cheaters and non-cheaters, many people cheating just a little, or both. Whereas no participants in the control condition reported perfect scores (a “10”) on Test 1, 44% of participants in the answers condition did. However, even excluding perfect scores, Test 1 scores were higher in the answers than the control condition (6.20 vs. 4.51). This suggests many people cheating just a little, consistent with Mazar et al. (2008) theory of self-concept maintenance, which posits that people avoid negative self-signals by cheating only within an acceptable range.

#### Behavioral Self-deception

We expected that if participants in the answers condition were self-deceived, their predictions for subsequent tests would be higher than their actual scores; we expected this gap to be highest on Test 2—immediately after participants had cheated to achieve a high score on Test 1—and to decline over time. We did not expect participants in the control condition—who were not given the opportunity to cheat—to show a gap between their predictions and actual performance on Tests 2 through 4.

A paired *t*-test confirmed that Test 2 predictions exceeded Test 2 scores for participants in the answers condition, *t*(35) = 3.67, *p* = 0.001, *d* = 0.73 (Table 1) reflecting self-deception: despite having had the chance to examine the questions on Test 2 and confirm no answers were included, participants in the answers group expected to perform better than they did. Their surprisingly low scores on Test 2 did not eliminate their self-deception: their predictions for Test 3 were also significantly higher than their Test 3 scores, *t*(35) = 2.52, *p* = 0.02, *d* = 0.35 (Table 1). Only after scoring below their expectations on both Tests 2 and 3 did self-deception decay completely: predictions for Test 4 were not significantly higher than actual scores, *t*(35) = 1.13, *p* = 0.27, *d* = 0.20 (Table 1).

By contrast, predictions did not differ significantly from scores for participants in the control group for any of the three tests: Test 2 [*t*(34) = 1.36, *p* = 0.18], Test 3 [*t*(34) = 0.95, *p* = 0.35], Test 4 [*t*(34) = 0.67, *p* = 0.51] (Table 1). The lack of overprediction in the control group also indicates the inflated predictions of participants in the answers condition are not related to mere overconfidence: overconfidence would suggest people might generally inflate their predictions (Moore and Healy, 2008), but this pattern was not observed.

#### Dispositional Self-deception

**FIGURE 1:**
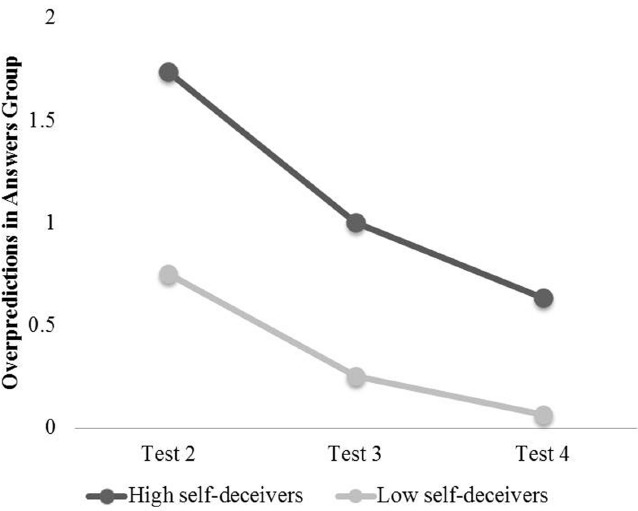
**Overpredictions on Tests 2-4 by high and low self-deceivers in Study 1**.

We also explored whether the general tendency to self-deceive would relate to the decay in the observed prediction-performance gaps. Self-Deceptive Enhancement was indeed correlated with overpredictions on the second test (*r* = 0.40, *p* = 0.02) in the answers condition, but not the control condition (*p* = 0.79). A median split on Self-Deceptive Enhancement revealed that high self-enhancers were driving the self-deceptive predictions observed in the answers group, and that their bias was strong even in predictions for Test 3. High self-deceivers significantly overpredicted their scores on Test 2 [6.58 vs. 4.84, *t*(18) = 3.07, *p* = 0.007, *d* = 0.93] as well as Test 3 [5.95 vs. 4.95, *t*(18) = 2.73, *p* = 0.01, *d* = 0.57], but eventually even this group tempered their expectations to conform to reality, more accurately predicting their scores on Test 4 [5.74 vs. 5.11, *t*(18) = 1.23, *p* = 0.24]. Low self-deceivers in the answers group, on the other hand, did not show significant differences between any of their predictions and subsequent scores (all *p*’s > 0.10). This pattern of results is shown in Figure 1. As expected, Impression Management showed no significant relationship to overpredictions in either the answers or the control group (all *p*’s > 0.10), suggesting that the overpredicting observed here does not derive merely from a strategy to impress others such as the experimenters. For the answers group, we also compared Self-Deceptive Enhancement of those reporting perfect scores (likely cheaters) to those scoring lower; although the sample size was small and the observed difference not significant, those reporting perfect scores showed directionally higher Self-Deceptive Enhancement [7.19 vs. 6.20, *t*(34) = 0.64, *p* = 0.52]. Note that the self-deception observed here is not complete: participants in the answers condition do predict lower scores on Test 2 than they received on Test 1. These results suggest that rather than witnessing complete self-deception, we observe a self-deceptive miscalibration that then diminishes even more in the face of feedback.

These results demonstrate that self-deceivers come to terms with reality only when faced with repeated exposure to counterevidence against their preferred beliefs—for these participants, scoring lower on multiple tests they could not cheat on—and do so eventually rather than immediately. This pattern is most striking for those with a dispositional tendency toward self-enhancement.

## Study 2: The Revival of Self-deception

Study 1 showed that when a single episode of cheating results in superior performance, it can lead to self-deception, but that repeated corrective feedback diminishes self-deception over time. However, in addition to providing evidence of a person’s true abilities, life also offers repeated temptations to engage in questionable behavior, and thus repeated opportunities to self-deceive. Could later opportunities to cheat reinstate self-deception, overwhelming the educational effect of corrective feedback?

In Study 2, after some participants had cheated on Test 1 and had then taken Test 2 without an answer key and received legitimate feedback, we gave them a second chance to cheat by providing them with answers for Test 3. We predicted that those with the answer key for Test 3 would cheat again, and that their inflated scores would revive self-deception, evidenced by inflated predictions of their scores on Test 4.

### Materials and Methods

#### Participants

One hundred forty-eight student and community member participants (68 male, Mage = 23.0, SD = 2.10) from the paid subject pool of a large, northeastern university were paid $20 to complete this experiment as the first of a series of unrelated studies during a 1-h lab session. Participants also had the opportunity to earn performance-based bonus pay. Sample size was determined by laboratory capacity, and privacy dividers separated participants from one another.

#### Design and Procedure

The design, procedure, and incentives in Study 2 were similar to those in Study 1. Briefly, participants took four tests and earned $0.25 for every correct answer. After each test was completed and scored, and after they had seen the answers, they looked over the next test, predicted their score, and completed the test. The only difference between Study 2 and Study 1 was that participants in the answers condition had an answer key at the bottom of Test 3 as well as Test 1.

### Results and Discussion

#### Cheating

We predicted that participants in the answers condition would cheat when they had the opportunity, reporting higher scores than the control group. This was true in both cases in which they had the answer key, Test 1 [*t*(146) = 8.07, *p* < 0.001, *d* = 1.33] and Test 3 [*t*(146) = 8.79, *p* < 0.001, *d* = 1.46] (Table 2).

**Table 2:** **Study 2 scores and predictions**.

		**Test 1**	**Test 2**	**Test 3**	**Test 4**
Answers	Prediction		6.06	6.95	5.75
	Score	7.65*	5.55	7.46*	5.28
Control	Prediction	4.97	4.51	4.79
	Score	5.03	5.34	4.68	4.88

*Answer key available, cheating possible.

#### Self-deception

We also predicted, as in Study 1, that participants who had the opportunity to cheat on Test 1 would self-deceive: we expected their Test 2 predictions to be higher than their actual scores. A paired *t*-test confirmed that Test 2 predictions were indeed higher than Test 2 scores for participants in the answers condition [*t*(79) = 2.69, *p* < 0.01, *d* = 0.24] but not for those in the control condition, who predicted marginally lower scores than they achieved [*t*(67) = 1.87, *p* = 0.07] (Table 2).

When participants in the answers condition predicted their Test 3 scores, they did so with the knowledge of the answer key at the bottom of that test. We had no specific hypothesis regarding these predictions because we were interested in determining how cheating on Test 3 might influence their predictions for Test 4. We found Test 3 predictions for those in the answer key group were lower than the scores [*t*(79) = 2.59; *p* = 0.01, *d* = 0.23], whereas predictions for those in the control condition did not differ from the scores [*t*(67) = 0.91; *p* = 0.37] (Table 2).

Our key hypothesis in this study was that participants in the answers condition would reengage in self-deception after the second opportunity to cheat, and would predict unrealistically high scores on Test 4. As expected, they did so [*F*(79) = 6.73, *p* = 0.01, *d* = 0.23], whereas those in the control group did not predict unrealistically high scores [*F*(67) = 0.12, *p* = 0.73] (Table 2). A second opportunity to cheat appears to have reinstated self-deception, overcoming any learning from the unbiased feedback on Test 2.

## General Discussion

One might expect people who cheat on tests—or insider traders—to feel worse about their abilities as a result of their questionable behavior. After all, if they had been more talented, they would have had no reason to cheat. However, when self-deception is possible, ethics can fade (Tenbrunsel and Messick, 2004). People tend to focus on the positive outcome of their cheating and neglect the unsavory process that led to it.

Although the construct of self-deception has a long history in psychology, the nature of the process by which self-deception takes place is still subject to debate (Audi, 1997; Mele, 2010; Bandura, 2011; McKay et al., 2011; von Hippel and Trivers, 2011). In these two studies, we showed that though self-deception does occur rapidly, there is some decay over time, suggesting that self-deception may provide temporary boosts to the self-concept but that these boosts may be relatively short-lived given corrective feedback from the environment (Study 1). Additionally, Study 2 demonstrates that sensitivity to feedback depends on the extent to which it enables self-deception; feedback bolstering motivated beliefs in superior abilities seems to be given more weight than feedback about actual abilities. As a result, it appears as though people are vulnerable to serial self-deception, awaiting opportunities to inflate their self-views and only grudgingly adjusting them downward. Study 1 demonstrates that inflated predictions of subsequent performance in the answers group correlate with general self-deceptive enhancement, and have suggested that these results suggest that participants engage in self-deceptive miscalibration. Future research might disambiguate total self-deception from general miscalibration by comparing predictions of own scores to predictions of others’ scores, allowing an assessment of whether people demonstrate self-deceptive miscalibration only when they are the focal actor, or whether even observing others induces miscalibration.

In our studies, we explored self-deception using a specific set of tasks similar to test situations in which students might have the opportunity to cheat. Although our focus was the impact of self-deception on people’s beliefs about their future performance, self-deception in similar contexts might also affect subsequent behavior. It could, for example, lead students to spend less time preparing for future tests, thus reducing their learning as well as hampering their future performance. It might also increase the likelihood of cheating again, by allowing people to feel good about themselves and their abilities when they cheat (and then self-deceive). Future research is needed to examine these negative behavioral consequences of self-deception, not only in the context of academic cheating but also in the many situations in which people inflate their performance by cheating and then deceive themselves about why they did so well.

### Conflict of Interest Statement

The authors declare that the research was conducted in the absence of any commercial or financial relationships that could be construed as a potential conflict of interest.
